# 

**Published:** 1911-03

**Authors:** 


					﻿Vol. XXVI.
MARCH, 1011/
No. 9.
TEX AIS
MEDICAL
JOURNAL
“Red Back”
PUBLISHED AT AUSTIN, TEXA^CT^^g^^u^^
F. E. DANIEL, M. D., - - - E^itopran^; Proprietor?'^.
PRINTED BY THE VON BOECKMANNINES’C’Q- Q,
Subscription, SI.00 a Year, in Advance. -	-	-	- Single Copies, 10 Cents
Entered at the Postoffice at Austin, Texas, as second class mail matter.'
				

